# Investigation of the Influence of Viscoelastic Behaviour on the Lifetime of Short Fibre Reinforced Polymers

**DOI:** 10.3390/polym12122874

**Published:** 2020-11-30

**Authors:** Gabriel Stadler, Andreas Primetzhofer, Michael Jerabek, Gerald Pinter, Florian Grün

**Affiliations:** 1 Montanuniversität Leoben, Chair of Mechanical Engineering, Franz-Josef-Strasse 18, 8700 Leoben, Austria; Gabriel.Stadler@unileoben.ac.at (G.S.); florian.gruen@unileoben.ac.at (F.G.); 2 Polymer Competence Center Leoben, Roseggerstrasse 12, 8700 Leoben, Austria; Gerald.Pinter@unileoben.ac.at; 3 Borealis Polyolefine GmbH, St.-Peter Strasse 25, 4021 Linz, Austria; Michael.Jerabek@borealisgroup.com; 4 Montanuniversität Leoben, Chair of Polymer Testing, Otto-Glöckel-Strasse 2, 8700 Leoben, Austria

**Keywords:** polypropylene, fatigue, long-time material behaviour, creep, time to failure, testing method

## Abstract

Short fibre reinforced polymers are getting more important for structural applications. Becasue of lightweight actions, components are designed for a specific application and lifetime. The bearable numbers of cycles can be estimated using material data and models for the consideration of influence factors. Further static loadings affect material behaviour, which influences the component lifetime. Commonly used models are not able to capture these effects. Therefore, material tests, with different load sequences, on 40% short glass fibre reinforced polypropylene have been performed. These sequences are combinations of cyclic and static loads at different, defined levels. Our research shows a lifetime elongation or reduction of a polymer, depending on the amount of static load time and quantity. For a certain stress level, the time to failure can be elongated or shortened more than a decade by another stress level, as compared to pure cyclic load. Additionally, the stiffness development of the composite is investigated in order to capture the damage course. Accordingly, these effects needed to be considered in lifetime prediction.

## 1. Introduction

Official regulations force automotive manufacturers to reduce greenhouse gas emissions. This is realized with improved drive technologies and reduced structure weight using application-oriented materials. Lightweight materials, like short fibre reinforced polymers (sfrp) for structural applications, obtain increased importance to reach the proceeded goals. Because sfrp components are manufactured by the injection moulding process, shape optimized components can be realized. In order to use the material potential for different specific applications, the fatigue limits of the used materials must be known. Usually, fatigue tests are performed to obtain the data for a lifetime estimation of short fibre reinforced polymers [1]. These calculations consider several influence factors, like local fibre orientation [2,3], temperature, and notches [4]. Their analysis did not take static loads into account for the lifetime prediction. Research has tended to focus on fatigue behaviour of “engineering polymers” or “high performance polymers”, rather than “standard polymers” (classification in [5]). For cost reasons, fibre reinforced “standard polymers” with different types and contents of fibres are becoming increasingly important for structural applications. In order to generate long-time data and knowledge about the material behaviour of “standard polymers”, many attempts have been made [6,7,8,9]. Moreover, built-in components are also loaded with static stress during a conventional use, which should be investigated and considered in lifetime estimation. Because static loads also influence the material behaviour, especially at standard polymers, long time tests deliver data for static material behaviour. If a rupture occurs, the static failure limit, which is known as creep strength, is reached [10,11]. Test sequences with static and fatigue blocks have been developed in order to evaluate the influence of static load on fatigue. For this methodology, the stress ratio as well as the static and cyclic block times have been varied. Moreover, different static stress levels between the cyclic loads have been investigated. The results show an elongation or reduction of the lifetime, depending on the constant load level. Hence, this needs to be considered in lifetime assessment.

### 1.1. Theory

Polymers, especially “standard polymers”, show a pronounced viscoelastic behaviour with mechanical loadings. Several authors [9,10,12,13] pointed out this behaviour also on reinforced polymers. Different testing methods are used to obtain stress-strain data for specific model parameters. Static tests, like creep or recovery tests, are settled standard test methods for long time behaviour investigation [9]. These methods can be accelerated using different temperature levels [14]. The result is a master curve with a time depended modulus development for a certain polymer [15]. These curves serve as basis for long-time parameter deduction, e.g., the creep modulus ($E_{c}$, Equation (1)). In this chapter, the standard meaning of σ is used for stress, ϵ for strain, and *t* for time [16].

(1) $$E_{c}\left(t\right)=\frac{\sigma }{\epsilon (t)}$$

The limit for static tests is defined with a creep strength for a certain creep time. Creep rupture tests are performed to reach this strength for a certain stress level. One result of these tests is also a drop in the creep modulus during the entire test period [17].

**Figure 1:**
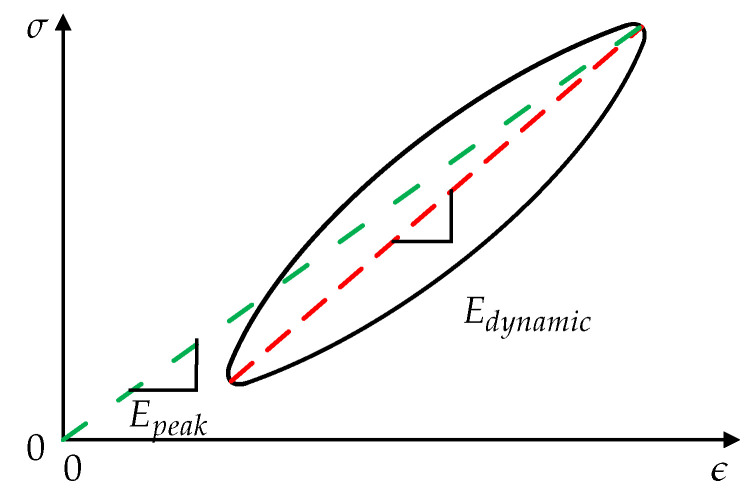
Determination dynamic modulus and peak modulus.

Different types of moduli (e.g., creep, peak, and dynamic modulus) are taken into account to describe the viscoelasticity of polymers and polymer composites during a service time. For these investigations, two parameters (in our case moduli) that define the position and location of the hysteresis loop in a stress-strain diagram, are used. One of these parameters is the peak modulus, also known as secant modulus [18,19]. This modulus is defined with the maximum stress $\sigma_{max}$ and the related strain $\epsilon (\sigma_{max})$ in a loop according to the origin of the diagram (Equation (2), Figure 1).

(2) $$E_{peak}=\frac{\sigma_{max}}{\epsilon (\sigma_{max})}$$

The second parameter, the dynamic modulus, is defined with maximum and minimum hysteresis stress and the related strains (Equation (3), Figure 1) [19].

(3) $$E_{dynamic}=\frac{\sigma_{max}-\sigma_{min}}{\epsilon \left(\sigma_{max}\right)-\epsilon \left(\sigma_{min}\right)}$$

The calculated moduli equal the slopes of the hysteresis curves and indicators for viscoelasticity and damage.

The resulting damage factor is calculated by the difference of the dynamic and peak modulus (Equation (4)).

(4) $$D\left(t\right)=E_{dynamic}\left(t\right)-E_{peak}\left(t\right)$$

This factor gives the stiffness drop, caused by damage. Accordingly, this factor is named “damage” in this paper.

### 1.2. Viscoelasticity

Viscoelastic behaviour is determined with creep or/and recovery tests and described by mathematical models. For the long time description of the creep modulus ($E_{c}$), a series of exponential terms, so-called “Prony-Series” can be used (Equation (5)). The parameter $E_{0}$ stands for the initial modulus, $\tau_{i}$ is the creep time for every creep term and *t* is the test time.

(5) $$E_{creep}\left(t\right)=E_{0}\cdot \left(1-\sum_{i=1}^{n}p_{i}\cdot \left(1-e^{-t/\tau_{i}}\right)\right)$$

This parameter also depends on the stress level of the constant load. Further, stiffness loss, as represented by the creep modulus, also includes creep damage. Using this loss for cyclic and creep load as an indicator for damage is proposed for cellular composites [20]. Moreover, Guedes et al. [21] already noted that there is a relation between static and cyclic failure, depending on the failure criterion. Based on these informations, the moduli may also be an indicator for the damage of sfrp.

## 2. Materials and Methods

**Figure 2:**
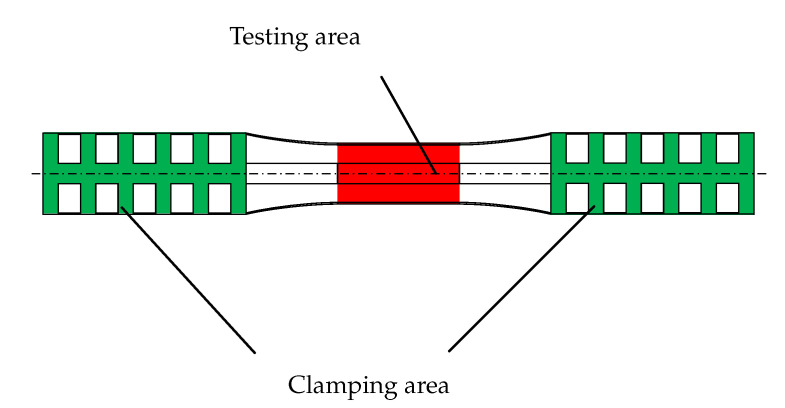
Specimen for the tests.

The investigated material is a polypropylene with a glass fibre content of 40% (PP-GF40). The injection moulded specimens for these tests are “rotating bending specimens”. This specimen type has been used in the past and is appropriate for fatigue tests; the exact geometry can be seen in [22] (Figure 2).

The area of interest, the parallel part in the specimens centre (“testing area”), is taken into account for further investigations.

### 2.1. Experimental

**Figure 3:**
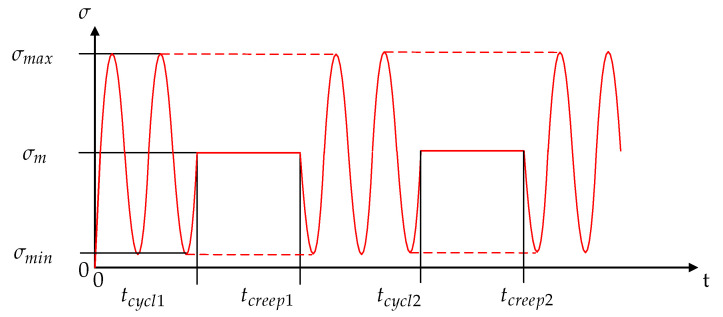
Block test sequence.

Different types of test sequences have been performed to obtain the fatigue and long-time behaviour of the polymer composite. Fatigue tests on a servo-hydraulic testing machine (MTS 810, MTS Systems GmbH, Berlin, Germany) at stress ratio of R = 0.1 and frequency of 3 Hz serves as a reference. Two extensometers (MTS, reference length 10 mm, mounted on the opposite) captured the local strains (mean value of the extensometers) of the specimen’s testing area. The environmental conditions for these tests were set to room temperature and 50% relative humidity. To obtain the influence of static loads on the fatigue behaviour, modified test sequences were performed. This methodology is a combination of alternating cyclic and static loads. While the cyclic part of the test block is kept at a constant stress ratio of R = 0.1, the static levels have been changed between the sequences. The load level remains at maximum ($\sigma_{max}$), mean ($\sigma_{mean}$), or minimum ($\sigma_{min}$) of the cyclic stress for one test sequence. A similar test methodology has been used for long fibre reinforced composites [23], but only with constant load at maximum stress. The relationship between the static and cyclic load times varies from 0% (pure cyclic load), 25%, 50%, up to 75% (Figure 3). Consequently, an influence of creep damage with increased creep duration can be investigated.

### 2.2. Analysis

The S/N curves of the cyclic and the combined tests are evaluated according to ASTM E739-91 [24]. Further, the development of the peak, dynamic, and creep modulus of the tests are analysed. For this purpose, the tests are separated into cyclic and static parts of the test block. The analysis for each modulus is performed by stringing the cyclic parts together. This approach is also done with the static parts of the tests. The results of these tests are the moduli developments for the cyclic and static loads until breakage.

## 3. Results

**Figure 4:**
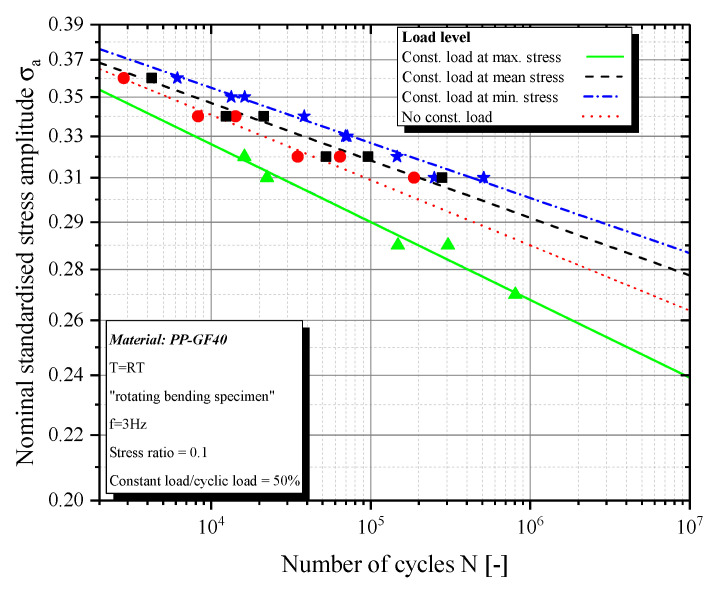
S/N-Curvefor various stress levels and 50% creep share.

Our investigations of the tests results are separated into two aspects, namely fatigue (number of cycles) and failure (total lifetime). This separation allows using pure fatigue and pure creep tests as a reference. The results show a main influence of constant load on the fatigue strength of sfrp. According to the reference fatigue tests at a stress ratio by R = 0.1, various stress levels affect the S/N curves, normalized to tensile strength, in different ways (Figure 4).

**Figure 5:**
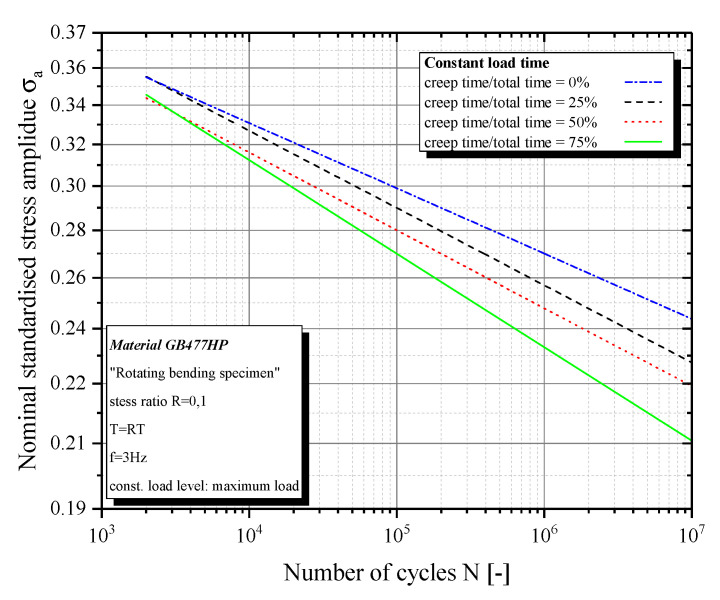
Influence of the constant load share on the lifetime.

The stress level at the stress maximum shortens the lifetime, while constant load at mean and minimum stress elongates the lifetime. This phenomenon is probably a result of the pre-strains from the cyclic parts. In the case of constant levels at mean or minimum stress, the pre-strains are higher than the creep strains at these stress levels that occurred by pure cyclic load. As a result, a relaxation of the strains occurs with the constant stresses. Consequently, this also affects the lifetime of the material. Because the strains at the beginning of the next cyclic block are lower than the mean strain from pure cyclic load, the material has time to reach the “regular” (position of the hysteresis loop with pure cyclic load) status. This means that cyclic creep has more effect on the mean strain than constant load at mean stress. The constant stress at the maximum level stretches the material further between the cyclic parts. As a result, the material gets a higher mean stress for the first cycles, which leads to a lifetime reduction. The strains at different stress levels can be shown by creep, dynamic, and peak modulus (Figures 9–11). Moreover, there is an influence of the creep share on the lifetime (Figure 5). It can be shown that an increase of the creep share at mean and minimum stress, also increases the lifetime of the specimen. The opposite effect is visible by a constant load at maximum stress, whereby a higher share of creep load shortens the lifetime (Figure 5) further.

**Figure 6:**
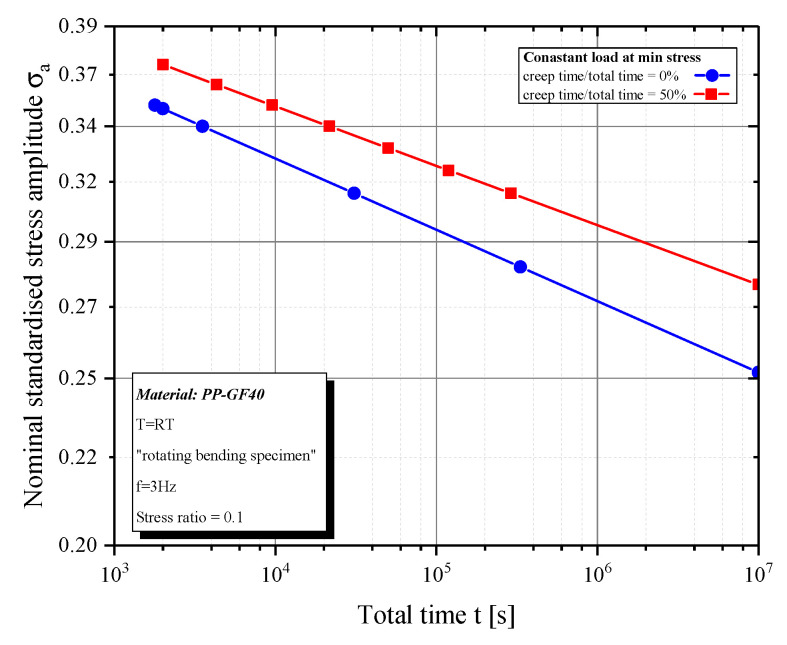
Time to failure for constant load at minimum stress.

By investigating the time to failure, it can be seen that there is a main influence depending on the load level of the constant stress. Keeping the creep load at minimum level, there are time to failure differences of factor 5.3 ($\sigma_{a}=0.33$) to 13.3 ($\sigma_{a}=0.27$), depending on the stress level (Figure 6). Because more static blocks are passed through at low stress levels, the influence on time to failure and also cycles to failure (Figure 5) is higher than with high constant loads.

**Figure 7:**
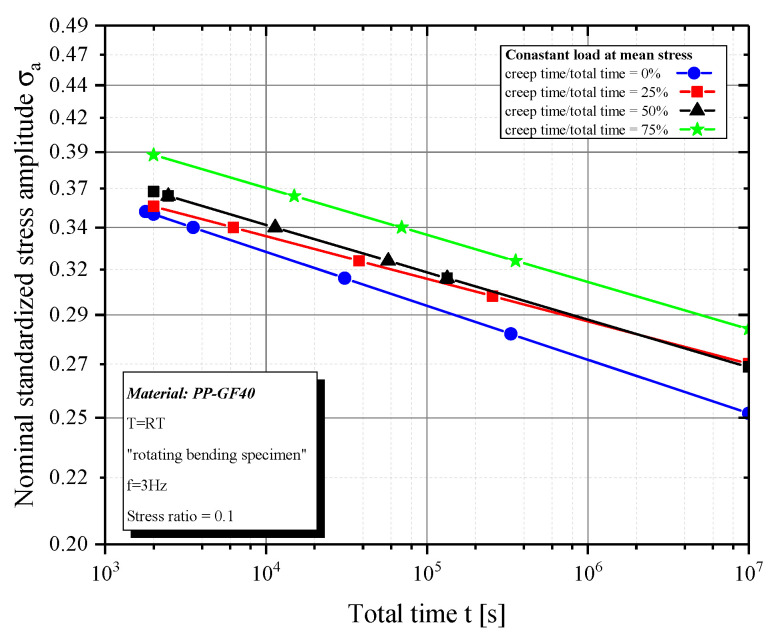
Time to failure for constant load at mean stress.

When comparing times to failure of 50% constant load tests with cyclic tests, there is a difference of a factor of 3.2 ($\sigma_{a}=0.33$) to 5.6 ($\sigma_{a}=0.27$) (Figure 7). This result suggests a lower relaxation by the mean stress level when compared to the minimum stress level.

**Figure 8:**
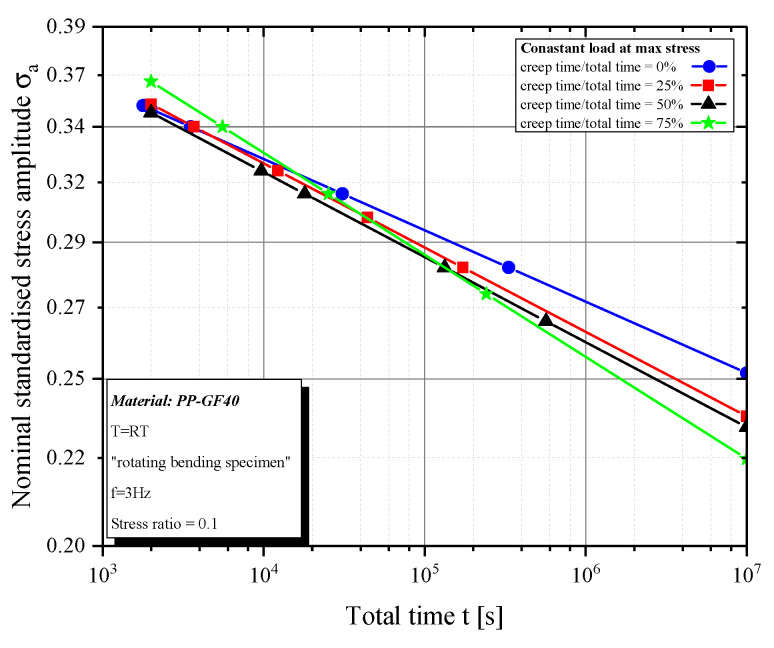
Time to failure for constant load at maximum stress.

The time to failure is nearly independent of the block time with a constant load level at maximum stress (Figure 8). However, there is a main difference on the number of cycles (Figure 5).

**Figure 9:**
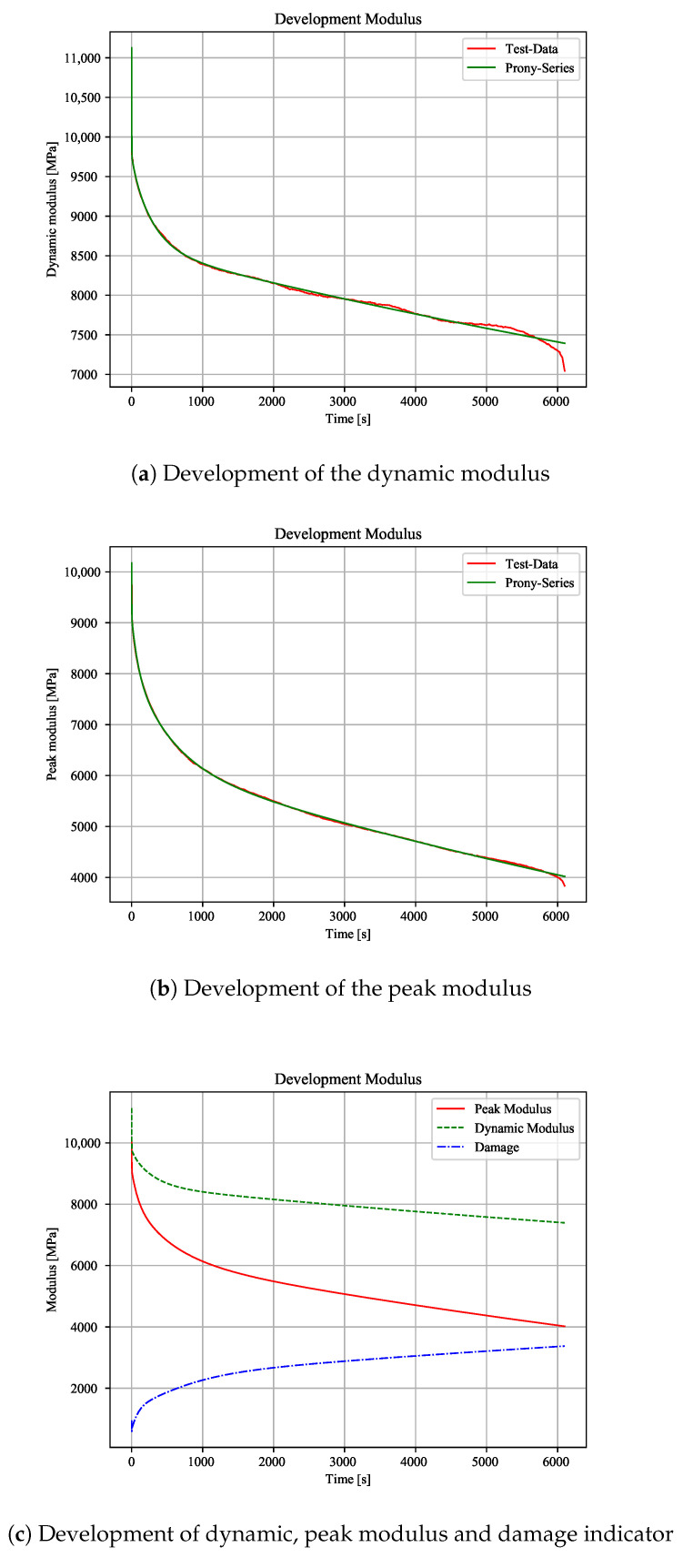
Development of moduli with cyclic load (stress amplitude 0.34).

Therefore, it can be shown that a constant load at maximum stress causes a nearly equivalent damage when compared to pure cyclic load in a certain time. Because a longer creep duration leads to higher initial strains (including creep damage) for the next cyclic load, the bearable number of cycles is reduced, depending on the static load share (Figure 5). It can be assumed that there will not be an effect of the frequency on the number of cycles if the temperature increase can be kept low. However, the frequency affects the moduli values and it needs further investigations. The results of the modulus analysis show an exponential drop of the moduli over the entire test time. Under pure cyclic load, the dynamic modulus steadily decreases throughout the test. This decrease can be well described with an exponential function, like the “Prony Series function” (Figure 9a).

**Figure 10:**
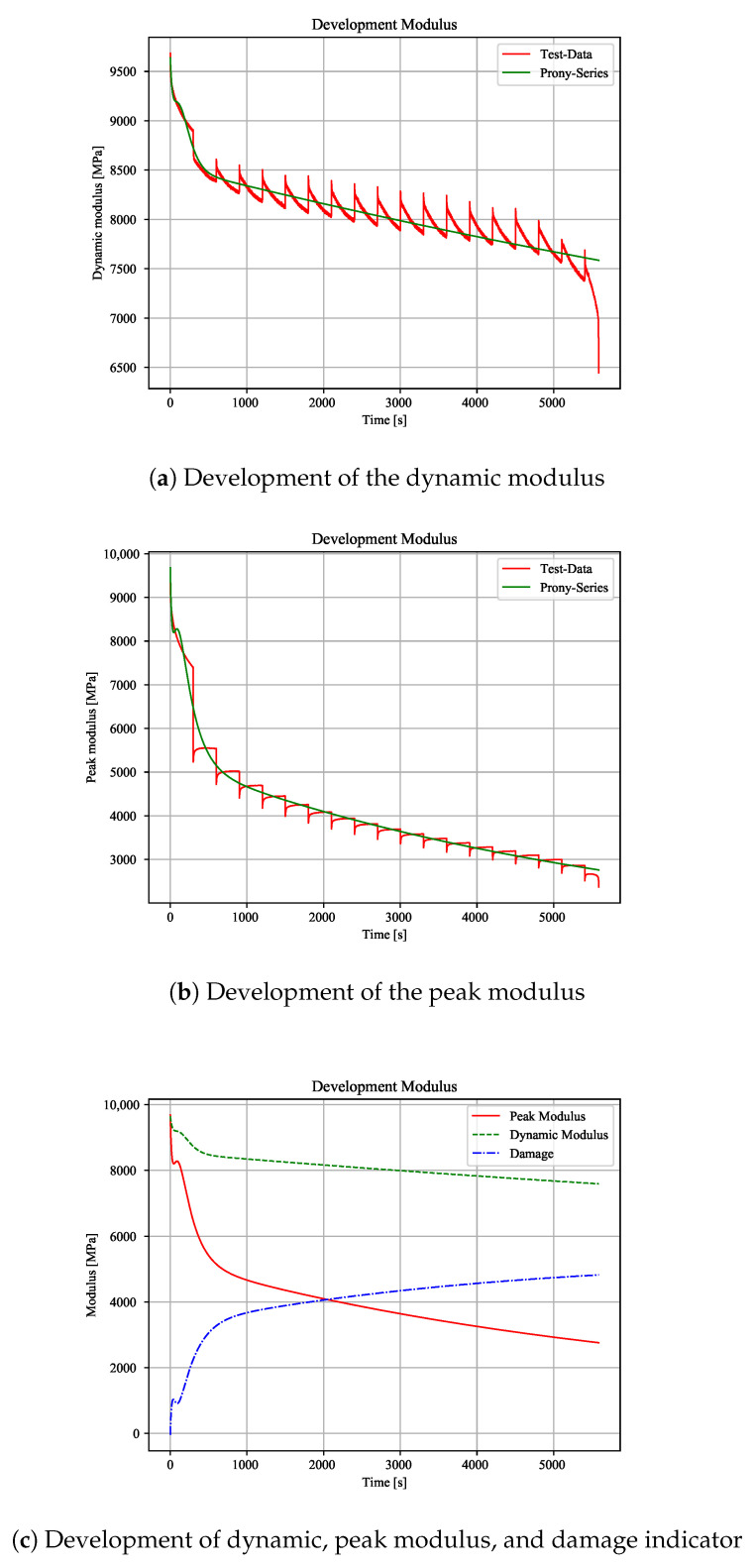
Development of moduli with cyclic load including static load blocks (stress amplitude 0.3, 75% constant load at maximum load level).

There is a decrease of the dynamic modulus by about 1/3 as compared to the initial value. Additionally, the peak modulus also drops exponentially. Thus, Prony Series are also used for this modulus (Figure 9b). It can be shown that there is an exponential damage by using the moduli difference as indicator (Figure 9c). Because the peak modulus includes cyclic creep, these results suggest a constant (cyclic) creep during the whole test. A similar course can be shown with combined tests. Because the cyclic parts are interrupted by static parts, the moduli show small prongs. For a maximum load and 75% creep share, this effect can be clearly shown. The dynamic modulus shows a major decrease during cyclic loading block (Figure 10a). The initial modulus values after a constant load level are higher than the last value of the previous cyclic block. This behaviour suggests a higher strain at the cyclic valley than with pure cyclic load, due to an over proportional material elongation.

**Figure 11:**
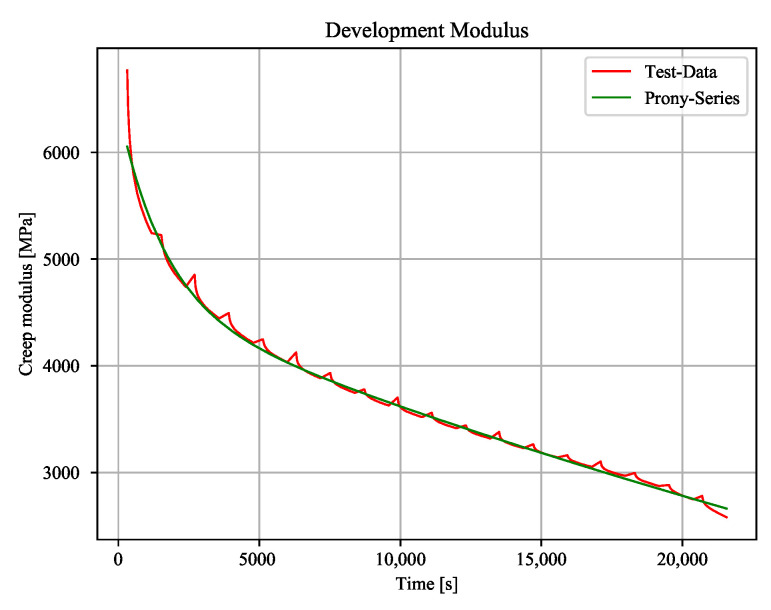
Development of the creep modulus.

The peak modulus shows small increases every static block. There, the maximum strain is relaxing after the constant stress block (Figure 10b). Therefore, the cyclic blocks have a relaxing effect on the material. Moreover, it can be shown that there is an higher increase of the damage indicator when compared to pure cyclic load (Figure 9c and Figure 10c). Because the resulting percentage modulus drops are independent of the creep stress level (Table 1), the increase of the damage is equal to pure cyclic load. When comparing the moduli from cyclic and combined tests, there is a nearly equal drop of the moduli for a certain time to failure. The creep modulus is dropping with every creep block due to increasing strains. Consequently, the initial strains for the next cyclic block is higher than strains from pure cyclic load (Figure 11). This effect switches to an opposite behaviour by reducing the constant load level.

The correlation between the modulus drop and lifetime of the specimen is summarised in Table 1. Mainly, the dynamic and peak modulus are the most interesting values, since they are compared to pure cyclic values. The values provide an indicator for the fatigue state of the polymer.

**Figure 12:**
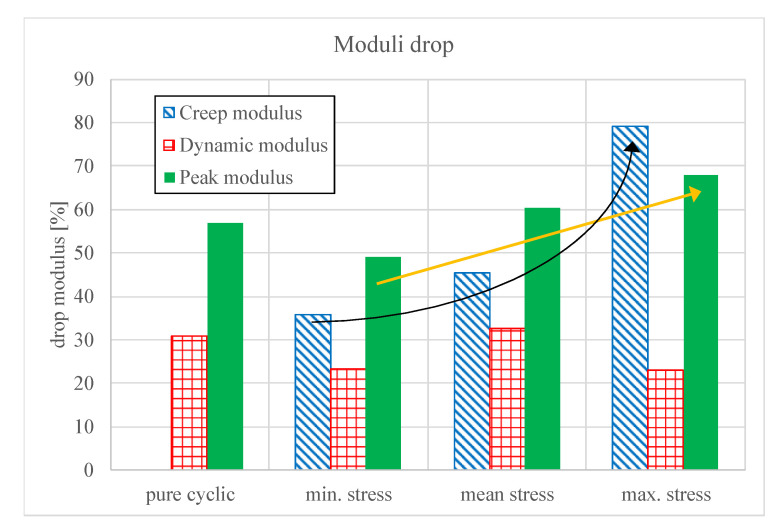
Drop of the moduli at different constant load levels.

The results show a correlation between the modulus drop and the S/N-curves. Cyclic tests serve as a reference with a dynamic modulus drop of 30.9% and peak modulus drop of 56.9%. Further, the peak modulus loss increases lineary with the constant load level, while the creep modulus loss rises exponentially (Figure 12).

## 4. Discussion

The study shows different phenomena concerning the moduli developments and the lifetime. Accordingly, the constant stress level (min, mean, max) has a higher influence on the creep modulus than on the peak or dynamic modulus. Consequently, the creep modulus is a useful indicator of the influence of the stress level on the lifetime. Whereas, the share of the constant loads at a certain stress level has less effect on the creep modulus drop. Keeping the constant load at mean stress, the modulus is increasing by a decreasing strain at this stage. This phenomenon is a result of the cyclic energy that is related to the constant strain level. A constant stress rate at maximum stress delivers a high creep energy level leading to a higher failure rate in the constant stress areas. By keeping the constant load at minimum or mean stress of the cyclic part, the creep energy is lower than the pure cyclic energy. Consequently, the strain decreases by these stress levels. Further investigation needs to be done in order to verify these phenomena. This study only captures one specific material and environmental condition. Further tests are necessary to obtain the influence of fibre content, fibre orientation, as well as temperature and humidity. Lower fibre contents and orientations will enhance the effect of static loads, which means that a lower lifetime would be reached with a constant load at maximum level. Additioanlly, higher temperatures shorten the lifetime and favours creep. The required tests and analysis have already started.

## 5. Conclusions and Outlook

Cyclic tests were performed to obtain reference fatigue data. To investigate the influence of constant load on the fatigue behaviour, cyclic tests with creep loads have been done. The shares of the constant loads have varied from 0%, 25%, 50%, and 75% of the total test time. Further, the stress level of the creep parts are set to the minimum, mean, and maximum of the cyclic stress. Our findings illustrate that there is a connection between the material behaviour and time to failure, depending on stress level. This study highlights the influence of static load on the lifetime of sfrp. These tests also reveal a correlation between the drop of creep, dynamic and peak modulus, and time to failure. The data and deducted parameters are applied (e.g., Prony parameters) for lifetime estimations including constant loads. Further data collection to determine exactly how the temperature and fibre orientation affects the creep and cyclic behaviour is required. In order to further our research, we plan to use this method for notched specimens and different stress ratios.

## Figures and Tables

**Table 1:** Percentage drop of the moduli (mean values of the tests)

Test Parameter	Dynamic Modulus	Peak Modulus	Creep Modulus
Cyclic	30.9%	56.9%	-
Creep at lower level	23.2%	49.0%	35.7%
Creep at mean level	32.6%	60.5%	45.3%
Creep at upper level	23.0%	68.0%	79.0%
